# Use of mobile and cordless phones and change in cognitive function: a prospective cohort analysis of Australian primary school children

**DOI:** 10.1186/s12940-017-0250-4

**Published:** 2017-06-19

**Authors:** Chhavi Raj Bhatt, Geza Benke, Catherine L. Smith, Mary Redmayne, Christina Dimitriadis, Anna Dalecki, Skye Macleod, Malcolm R. Sim, Rodney J. Croft, Rory Wolfe, Jordy Kaufman, Michael J. Abramson

**Affiliations:** 1 Centre for Population Health Research on Electromagnetic Energy (PRESEE), School of Public Health and Preventive Medicine, Monash University, 553 St Kilda Road, VIC 3004 Melbourne, Australia; 2 Australian Centre for Electromagnetic Bioeffects Research, School of Psychology, University of Wollongong, Wollongong, NSW 2522 Australia; 3 School of Health Sciences, Swinburne University of Technology, Hawthorn, VIC 3122 Australia

**Keywords:** Cognitive function, Cordless phone use, Mobile phone use, Primary school children

## Abstract

**Background:**

Some previous studies have suggested an association between children’s use of mobile phones (MPs)/cordless phones (CPs) and development of cognitive function. We evaluated possible longitudinal associations between the use of MPs and CPs in a cohort of primary school children and effects on their cognitive function.

**Methods:**

Data on children’s socio-demographics, use of MPs and CPs, and cognitive function were collected at baseline (2010–2012) and follow-up (2012–2013). Cognitive outcomes were evaluated with the CogHealth™ test battery and Stroop Color-Word test. The change in the number of MP/CP voice calls weekly from baseline to follow-up was dichotomized: “an increase in calls” or a “decrease/no change in calls”. Multiple linear regression analyses, adjusting for confounders and clustering by school, were performed to evaluate the associations between the change in cognitive outcomes and change in MP and CP exposures.

**Results:**

Of 412 children, a larger proportion of them used a CP (76% at baseline and follow-up), compared to a MP (31% at baseline and 43% at follow-up). Of 26 comparisons of changes in cognitive outcomes, four demonstrated significant associations. The increase in MP usage was associated with larger reduction in response time for response inhibition, smaller reduction in the number of total errors for spatial problem solving and larger increase in response time for a Stroop interference task. Except for the smaller reduction in detection task accuracy, the increase in CP usage had no effect on the changes in cognitive outcomes.

**Conclusion:**

Our study shows that a larger proportion of children used CPs compared to MPs. We found limited evidence that change in the use of MPs or CPs in primary school children was associated with change in cognitive function.

## Background

The use of mobile phones (MPs) and cordless phones (CPs) by young children has become common worldwide [1–6]. This has raised concerns regarding the potential health and psychological effects of MPs and CPs on the developing brains of children [7–11]. The World Health Organization (WHO) has identified research into children’s behavioral and neurological outcomes associated with radio-frequency electromagnetic field (RF-EMF) exposure as a high priority RF-EMF agenda [8].

The use of MPs or/and CPs in children has been associated with negative consequences in cognitive functioning [1, 12], including memory performance [11] and emotional and behavior difficulties [13]. However, there are few community-based epidemiological studies involving children that assessed effects of MP and/or CP use on cognitive functioning [1, 10–12, 14]. The findings of these studies are inconclusive [1, 11, 12, 14]. A recent Swiss study involving adolescents found a negative association between MP and CP use with figural memory [11]. The Amsterdam Born Children and their Development (ABCD) study showed inconsistent associations between MP and CP use and cognitive function [14]. Furthermore, our cross-sectional analysis of the ExPOSURE (Examination of Psychological Outcomes in Students Using Radiofrequency dEvices) study found little evidence for an association between the use of MPs and CPs and cognitive effects in a cohort of primary school children in Australia [10].

The aim of this prospective analysis of the ExPOSURE study data was to evaluate possible longitudinal associations between the use of MPs and CPs in a cohort of primary school children and effects on their cognitive function.

## Methods

### Study design and participants

A longitudinal study was undertaken among primary school children (the 4^th^ year) in Melbourne and Wollongong, Australia. Baseline (*n* = 619) and follow-up (*n* = 412) data were collected from 36 schools during November 2010–February 2012 and March 2012–March 2013, respectively. In Melbourne, schools were stratified as public (state), Catholic and private schools for recruitment. The schools located in central Melbourne regions and various adjoining regions were selected to be invited based on systematic sampling in each group of school type. Since the target number of schools was not recruited from central Melbourne and adjoining regions, schools outside of the originally selected regions were also invited to participate. The schools that agreed to participate were included in the study. The contact person from the schools nominated a class or classes to participate in the study. In Wollongong, all public and Catholic schools located within the greater Illawarra area were invited. Of them, all the schools that agreed to participate were included in the study. The schools that agree to participate then selected one or more grade four class(s) to be invited to take part in the study. Further details about sampling can be found elsewhere [10].

Written or verbal informed consent was obtained from the parent/guardian of each student, teachers of the participating classes, and principals of the participating schools. The study also received approvals from the Victorian Department of Education and Early Childhood Development, New South Wales Department of Education and Communities, Catholic Education Offices of Victoria and New South Wales, Monash University Human Research Ethics Committee and the University of Wollongong Human Research Ethics Committee.

The information on socio-demographics and that related to MP or/and CP use or ownership were collected at baseline and follow-up. Cognitive outcomes were also assessed at the two time points.

### Exposure assessment

The parents/guardians of participating children completed a modified and validated questionnaire from the Interphone study [15], which collected information on their children’s MP and CP use, such as average number of MP calls (made and received) weekly, duration of each (outgoing and incoming) MP call weekly, average number of text messages or SMS (sent and received) weekly, average number of CP calls (made and received) weekly, and duration of each call on CP weekly. In addition, we also gathered socio-demographic information such as age, sex, country of birth, ethnicity (languages other than English spoken at home) and residential post code. The parents were also asked about their perception of their and their family’s health risk in relation to MP use. The children, assisted by research staff, completed a questionnaire about whether or not they owned or used a MP, laterality of MP use, handedness (right or left handed), and the amount of gaming and computer/Internet use.

### Outcome assessment

Cognitive outcomes were assessed with a computerized CogHealth™ test battery (CogState, Melbourne, AU, 2005) [1, 10, 12], and the Stroop Color-Word test [16].

*CogHealth™* evaluated signal detection (simple reaction), identification (choice reaction), one-back (working memory), one card learning (visual attention), Go/No-Go (response inhibition), and Groton maze learning (spatial and executive ability) [10]. Further details of testing administration are discussed elsewhere [10, 12]. The total number of errors for the spatial problem solving function, and response times (ms) and accuracy (%) for the rest of the CogHealth™ cognitive tests were assessed.

*The Stroop Color-Word test*: This test assessed the ability to name colors and words that are presented in conjunction with interfering stimulus characteristics (e.g., naming the written word ‘red’, presented in blue hue) [17]. The task has four sub-tests, two to provide baseline information (no interference), and two interference conditions [1, 12]. These tests measured response time for each form and time ratios were subsequently estimated. Further details of the test can be found in the literature [1, 16].

### Statistical analyses

Descriptive analyses were performed for socio-demographics and MP and CP use. The descriptive analyses for exposure measures, CogHealth™ tasks, Stroop Color-Word test, and regression analyses were performed for the students taking part both in baseline and follow-up (*n* = 412). Socio-economic status (SES) was estimated from socio-economic indexes for areas in accordance with children’s residential postal codes [18].

RF-EMF exposure measures were the total number of voice calls (made and received) weekly for MP and CP separately. We used reported numbers of MP and CP calls as the proxies for MP and CP exposures, respectively [1, 10, 12], because they may be more accurate in ascertaining the phone use compared to the ‘duration of calls’ [19, 20]. We did not consider SMS use as an exposure, firstly because RF-EMF exposure to the head due to SMS would be very low, and secondly because SMS use was very low in this age group. MP use was low in both surveys. For the MP/CP users, the numbers of reported voice calls weekly on MPs and CPs were low (i.e., median at baseline = 2), with no use of MP/CP represented as zero calls. Therefore, the exposure metrics at baseline and follow-up were further classified in terms of the three groups: no use (‘none’), ≤ 2 voice calls weekly on MP/CP (‘some’), and > 2 voice calls weekly on MP/CP (‘more’). Cognitive parameters were summarized across these three groups. The mean response time of each test was log_10_ transformed and the square root of each accuracy score was arcsine transformed [1, 12]. The Stroop Color-Word test data were analyzed by comparing the time ratios of response times (in seconds) of form B and form A [i.e., (B-A)/A], and those of form D and form C [i.e., (D-C)/C].

Multiple linear regression models with robust standard errors, allowing for clustering of students within schools, were used to assess the association between the change in cognitive outcome (follow-up minus baseline) in children who increased their MP (or CP) weekly calls from baseline to follow-up, and those who did not increase their MP (or CP) usage. The models were adjusted for age at baseline, sex, ethnicity, SES (classified into quintiles), lag time between baseline and follow-up, handedness and total screen time weekly (i.e., gaming and computer/Internet use).

Changes in MP and CP voice calls weekly from baseline to follow up were dichotomized as follows: 0 = decrease or no change in number of MP and CP voice calls weekly, or 1 = increase in number of MP and CP voice calls weekly. The models also considered the potential interaction between gender and MP and CP use in an exploratory analysis. Seven children with attention deficit hyperactivity disorder were excluded from the regression analysis. For all analyses, *p* ≤0.05 (two-sided) was considered as statistically significant. Data analysis was performed with STATA ver13.1 (StataCorp, College Station, TX, USA) or SPSS (version 22, IBM Corp, Armonk, NY, USA).

## Results

### Descriptive data

Table 1 shows socio-demographic characteristics, and MP and CP use of the children taking part in baseline and follow-up. Of 619 baseline participants, 412 (66.5%) took part in the follow-up study. The mean (± SD) time lag between the baseline and follow-up studies was 12.4 ± 2 months. Most of the participants at baseline and follow-up were from high socio-economic areas. Ethnicity (language other than English spoken at home) and country of birth (Australia) of children was similar at baseline and follow-up. The handedness also remained largely unchanged from baseline and follow-up – right (87%), left (10.3%), no preference (2.2%) [baseline], and right (86%), left (10%) and no preference (~4%) [follow-up]

**Table 1:** Socio-demographic, mobile and cordless phone use characteristics of children

Variables	Baseline [*n* = 619]	Follow-up [*n* = 412]
Age (mean ± SD)	10 ± 0.4 years	11 ± 0.5 years
Sex	53% (*n* = 329) girls	55% (*n* = 227) girls
Ownership/use of a MP (parent’s response)	31.0% (*n* = 187)	43.3% (*n* = 168)
Ownership/and use of a MP (children’s response)	57.3% (*n* = 353)	67.9% (*n* = 279)
Laterality of MP use	Right: 68.2% (*n* = 262) Left: 11.5% (*n* = 44) Both: 18.5% (*n* = 71)	Right: 74.2% (*n* = 250) Left: 10.7% (*n* = 36) Both: 14.0% (*n* = 47)
Use of a CP at home	76% (*n* = 470)	76.2% (*n* = 314)
Average duration per call (minutes) weekly	Median (25^th^, 75^th^ percentiles)	Median (25^th^, 75^th^ percentiles)
A call dialed on MP	2 (1, 3)	2 (1, 3)
A call received on MP	1.5 (0, 3)	1.5 (0.5, 3)
Call (dialed & received) on CP	3.5 (2, 5)	4 (2, 5.5)

According to the parental responses, nearly 31% of children at baseline and 43% at follow-up owned or used a MP. In contrast, 57% of children at baseline and 68% at follow-up reported having owned or used a MP. The use of a CP at home was reported for 76% of the children both at baseline and follow-up.

Table 2 shows MP and CP calls, MP SMS, and MP and CP use for the children who took part both in baseline and follow-up. Overall, weekly MP voice calls and SMS significantly increased (*p* < 0.001 for both), whereas weekly CP voice calls remained unchanged (*p* = 0.26), from baseline to follow-up. For the MP/CP users, the median numbers of voice calls (made and received) weekly for MP and CP were 2 and 2 at baseline, and 2.5 and 2 at follow-up, respectively.

**Table 2:** MP and CP exposure measures [baseline & follow-up participants (*n* = 412)]

Total number of MP and CP calls/SMS weekly^a^	Baseline	Follow-up	
	Median (25^th^, 75^th^ percentiles)		^b^ *p*-value
MP voice calls (dialed & received)	2 (1,5)	2.5 (1,5.2)	**<0.001**
MP SMS (sent & received)	0.5 (0,4)	2 (0,7.5)	**<0.001**
CP voice calls (dialed & received)	2 (1,4)	2 (1,4)	0.26
MP and CP usage^c^	Proportion (number) of children		
MP voice calls			
No use	69.7% (*n* = 279)	57.6% (*n* = 220)	**<0.001** ^**d**^
Some use (≤2 calls weekly)	15.3% (*n* = 61)	22.0% (*n* = 84)	
More use (>2 calls weekly)	15.0% (*n* = 60)	20.4% (*n* = 78)	
CP voice calls			
No use	17% (*n* = 67)	20% (*n* = 76)	**0.04** ^**d**^
Some use (≤2 calls weekly)	47.5% (*n* = 186)	46% (*n* = 177)	
More use (>2 calls weekly)	35.5% (*n* = 139)	34% (*n* = 129)	

^a^Data included MP & CP users only, ^b^Wilcoxon signed-rank test, ^c^Data included both non-users and users, ^d^ Fleiss-Everitt Chi square test based on non-missing data, The bold numbers represent statistically significant assoctions

Of the parents taking part in both waves of surveys, 33.6% at baseline and 31% at follow-up considered that MPs posed low risk to them and their family’s health. Likewise, 21% at baseline and 22% at follow-up considered that MPs posed moderate risk. However 37% at baseline and follow-up did not know if MP use posed a health risk. Only a few parents (nearly 5% at baseline and follow-up) considered MPs to represent a high health risk. Five percent at baseline and follow-up also considered MP use to pose no health risk.

There were no significant differences between those who continued to participate (*n* = 412) and drop outs (*n* = 207) in age, sex, ethnicity, ownership or use of MP, laterality of MP use, handedness, total average number of calls (made and received) weekly on MPs, total average number of calls (made and received) weekly on CPs, total average number of SMS messages (sent and received) weekly on MPs. Nor were there differences in their baseline response times for the detection task, visual recognition and attention, and working memory; response time and accuracy for the identification task, or response inhibition task.

Compared to the children who were followed-up, the drop-outs were more likely to be in the lower two quintiles of SES, rather than the higher three quintiles of SES. The drop-outs also owned and used MPs and CPs less, had higher Stroop time ratios, response inhibition task, less accuracy in the Groton maze and detection tasks, visual recognition and attention and working memory (data not shown).

Tables 3, 4 and 5 summarize cognitive outcome data of the children who used MP and CP and took part in both waves of surveys. Table 3 compares data of CogHealth™ cognitive outcomes (untransformed) in those children who used MPs. Table 4 compares the data of the cognitive outcomes in those children who used CPs. Table 5 summarizes the cognitive outcome data of the Stroop Color-Word tests. These data show that response times tended to be faster and accuracy similar or better at follow-up compared to baseline in most cases.

**Table 3:** Descriptive statistics for mobile phone voice call use and CogHealth™ tasks [median (25^th^, 75^th^ percentiles)]^a^

Tests	Parameters	Baseline		Follow-up	
		MP voice calls (n)	Median (25^th^, 75^th^ percentiles)	MP voice calls (n)	Median (25^th^, 75^th^ percentiles)
Simple reaction time (Detection task)	Response time (ms)	None (276)	344 (302, 413)	None (243)	317 (288, 359)
		Some (59)	350 (297, 410)	Some (57)	315 (284, 342)
		More (57)	380 (321, 417)	More (83)	316 (282, 362)
	Accuracy (%)	None (278)	97 (92, 100)	None (243)	97 (95, 100)
		Some (61)	97 (92, 100)	Some (57)	97 (95, 100)
		More (58)	96 (83, 96)	More (83)	97 (93, 100)
Choice reaction time (Identification task)	Response time (ms)	None (277)	588 (524, 686)	None (243)	549 (481, 619)
		Some (61)	579 (518, 655)	Some (57)	525 (484, 577)
		More (58)	607 (550, 699)	More (83)	558 (493, 601)
	Accuracy (%)	None (278)	94 (86, 97)	None (243)	94 (88, 97)
		Some (61)	94 (88, 97)	Some (57)	94 (88, 97)
		More (58)	94 (86, 97)	More (83)	94 (89, 97)
One-back task (Working memory)	Response time (ms)	None (278)	965 (792, 1113)	None (243)	863 (707, 1035)
		Some (61)	1023 (793, 1164)	Some (57)	860 (741, 971)
		More (57)	976 (826, 1158)	More (83)	930 (748, 998)
	Accuracy (%)	None (278)	88 (77, 94)	None (243)	94 (86, 97)
		Some (61)	94 (81, 97)	Some (57)	92 (86, 97)
		More (58)	86 (69, 94)	More (83)	94 (82, 97)
One card learning task (Visual recognition memory & attention)	Response time (ms)	None (278)	1072 (885, 1322)	None (243)	1047 (854, 1273)
		Some (61)	1130 (881, 1387)	Some (57)	1004 (875, 1170)
		More (58)	1143 (887, 1293)	More (83)	1052 (936, 1225)
	Accuracy (%)	None (278)	59 (49, 66)	None (243)	64 (54, 71)
		Some (61)	57 (50, 64)	Some (57)	63 (53, 68)
		More (58)	58 (49, 64)	More (83)	64 (56, 70)
Go/NoGo (Response inhibition task)	Response time (ms)	None (276)	628 (539, 710)	None (243)	592 (505, 676)
		Some (60)	600 (557, 707)	Some (57)	549 (484, 628)
		More (58)	669 (599, 740)	More (83)	574 (516, 655)
	Accuracy (%)	None (277)	98 (94, 100)	None (243)	98 (96, 100)
		Some (61)	98 (94, 98)	Some (57)	98 (94, 98)
		More (58)	96 (91, 98)	More (83)	98 (96, 100)
Groton maze learning task (Spatial & executive ability)	Total number of errors	None (278)	69 (54, 86)	None (243)	55 (46, 67)
		Some (61)	65 (56, 76)	Some (57)	56 (49, 69)
		More (58)	72 (55, 89)	More (83)	59 (49, 72)

^a^ Statistics of children who took part in baseline and follow-up (*n* = 412)

**Table 4:** Descriptive statistics for cordless phone voice call use and CogHealth™ tasks [median (25^th^, 75^th^ percentiles)]^a^

Tests	Parameters	Baseline		Follow-up	
		CP voice calls (n)	Median (25^th^, 75^th^ percentiles)	CP voice calls (n)	Median (25^th^, 75^th^ percentiles)
Simple reaction time (Detection task)	Response time (ms)	None (66)	336 (306, 394)	None (76)	326 (294, 373)
		Some (183)	347 (299, 404)	Some (177)	325 (288, 357)
		More (135)	350 (307, 414)	More (123)	309 (279, 352)
	Accuracy (%)	None (67)	95 (89, 100)	None (76)	97 (95, 100)
		Some (185)	97 (94, 100)	Some (177)	97 (95, 100)
		More (137)	95 (87, 100	More (123)	97 (93, 100
Choice reaction time (Identification task)	Response time (ms)	None (67)	573 (526, 662)	None (76)	545 (496, 595)
		Some (185)	589 (521, 665)	Some (177)	559 (489, 611)
		More (137)	596 (536, 685)	More (123)	529 (476, 592)
	Accuracy (%)	None (67)	91 (85, 97)	None (76)	94 (91, 97)
		Some (185)	94 (86, 97)	Some (177)	94 (88, 97)
		More (137)	94 (86, 97)	More (123)	94 (88, 97)
One-back task (Working memory)	Response time (ms)	None (67)	958 (751, 1062)	None (76)	883 (729, 985)
		Some (185)	939 (793, 1114)	Some (177)	865 (715, 1022)
		More (137)	992 (818, 1132)	More (123)	865 (712, 1020)
	Accuracy (%)	None (67)	89 (74, 94)	None (76)	93 (86, 97)
		Some (185)	89 (77, 94)	Some (177)	94 (86, 97)
		More (137)	89 (74, 94)	More (123)	91 (84, 97)
One card learning task (Visual recognition memory & attention)	Response time (ms)	None (67)	1015 (827, 1274)	None (76)	1065 (940, 1345)
		Some (185)	1071 (887, 1312)	Some (177)	1042 (875, 1218)
		More (137)	1150 (874, 1340)	More (123)	1013 (862, 1224)
	Accuracy (%)	None (67)	56 (49, 65)	None (76)	64 (56, 70)
		Some (185)	60 (51, 66)	Some (177)	65 (56, 72)
		More (137)	59 (50, 65)	More (123)	61 (53, 69)
Go/NoGo (Response inhibition task)	Response time (ms)	None (66)	616 (558, 694)	None (76)	569 (517, 656)
		Some (185)	633 (541, 720)	Some (177)	580 (503, 662)
		More (136)	631 (565, 706)	More (123)	583 (503, 689)
	Accuracy (%)	None (67)	98 (94, 98)	None (76)	98 (96, 100)
		Some (185)	98 (94, 100)	Some (177)	98 (96, 100)
		More (136)	96 (94, 100)	More (123)	98 (94, 100)
Groton maze learning task (Spatial & executive ability)	Total number of errors	None (67)	65 (55, 84)	None (76)	56 (46, 67)
		Some (185)	69 (54, 80)	Some (177)	56 (46, 68)
		More (137)	68 (55, 84)	More (123)	58 (48, 70)

^a^ Statistics of children who took part in baseline and follow-up (*n* = 412)

**Table 5:** Descriptive statistics [median (25^th^, 75^th^ percentiles)] for the Stroop Color-Word test time ratios *vs* phone use type [CP (cordless phone) or MP (mobile phone)]^a^

Forms	Parameters	Exposure type	Baseline	Exposure type	Follow-up
		Groups (n)	Median (25^th^, 75^th^ percentiles)	Groups (n)	Median (25^th^, 75^th^ percentiles)
Stroop ratio ((B-A)/A)	Time ratio	MP voice calls		MP voice calls	
		None (276)	0.09 (0.02, 0.16)	None (218)	0.11 (0.03, 0.22)
		Some (60)	0.10 (0.02, 0.18)	Some (84)	0.13 (0.06, 0.22)
		More (60)	0.08 (0.003, 0.15)	More (78)	0.10 (0.05, 0.20)
		CP voice calls		CP voice calls	
		None (66)	0.07 (0.01, 0.14)	None (76)	0.11 (0.04, 0.20)
		Some (184)	0.89 (0.007, 0.17)	Some (177)	0.12 (0.04, 0.24)
		More (138)	0.11 (0.04, 0.17)	More (127)	0.10 (0.04, 0.20)
Stroop ratio ((D-C)/C)	Time ratio	MP voice calls		MP voice calls	
		None (276)	0.69 (0.53, 0.91)	None (218)	0.66 (0.52, 0.84)
		Some (60)	0.65 (0.52, 0.81)	Some (84)	0.60 (0.45, 0.81)
		More (60)	0.68 (0.59, 0.87)	More (78)	0.66 (0.50, 0.83)
		CP voice calls		CP voice calls	
		None (66)	0.60 (0.52, 0.77)	None (76)	0.56 (0.46, 0.71)
		Some (184)	0.70 (0.53, 0.93)	Some (177)	0.67 (0.53, 0.86)
		More (138)	0.68 (0.57, 0.89)	More (127)	0.66 (0.52, 0.85)

^a^ Statistics of children who took part in baseline and follow-up (*n* = 412)

### Association between change in cognitive function, and change in the use of mobile and cordless phones

Table 6 compares the change in cognitive outcome (follow-up minus baseline) between those who increased their MP/CP weekly calls from baseline to follow-up, and those who did not increase or reduce their MP/CP usage.

**Table 6:** Regression results comparing the change in cognitive outcome (follow-up minus baseline) between those who increased their MP (or CP) calls weekly from baseline to follow-up, and those who did not increase or reduce their MP (or CP) usage

	Difference in cognitive outcome means between those who increased MP (or CP) use and those did not increase or reduc their use				
	Voice calls on MP			Voice calls on CP	
Tests	Parameters	Estimate	95% CI	Estimate	95% CI
Detection task	Response time (ms)^a^	−0.008	−0.042, 0.027	0.007	−0.019, 0.033
	Accuracy (%)^b^	0.024	−0.036, 0.084	**−0.069**	**−0.127, −0.012**
Identification task	Response time (ms)^a^	−0.008	−0.034, 0.018	0.007	−0.011, 0.024
	Accuracy (%)^b^	0.012	−0.044, 0.067	−0.047	−0.097, 0.002
One-back memory task (Working memory)	Response time (ms)^a^	0.009	−0.018, 0.036	0.017	−0.006, 0.041
	Accuracy (%)^b^	0.014	−0.048, 0.076	−0.029	−0.077, 0.019
One card learning task (Visual recognition memory & attention)	Response time (ms)^a^	0.005	−0.033, 0.043	−0.0008	−0.034, 0.032
	Accuracy (%)^b^	−0.022	−0.054, 0.009	−0.011	−0.049, 0.026
Go/NoGo (Response inhibition task)	Response time (ms)^a^	**−0.030**	**−0.054, −0.006**	0.009	−0.013, 0.032
	Accuracy (%)^b^	0.011	−0.037, 0.059	−0.013	−0.060, 0.034
Groton maze learning task	Accuracy (total no. of errors)	**6.22**	**2.13, 10.31**	−2.77	−8.77, 3.24
Time ratio ((B-A)/A)	Response time (s)	**0.056**	**0.021, 0.090**	−0.028	−0.069, 0.013
Time ratio ((D-C)/C)	Response time (s)	−0.048	−0.127, 0.033	−0.003	−0.066, 0.059

Reference group: no change or decrease in total average number of MP or CP calls weekly (exposure dichotomized: “no change or decrease” and “increase” in total average number of MP and CP voice calls weekly)

Adjusted for age at baseline, sex, ethnicity, SES (classified into quintiles), lag time between baseline and follow-up, handedness, and total screen time weekly (gaming and computer/Internet use)

The bold numbers represent significant assoctions

^a^log_10_ transformed data, ^b^Arcsine transformed hit rate

Compared to the ‘no change or decrease’ group, the ‘increase’ in total average number of MP calls weekly group had significantly: i) larger mean reduction in response time for the Go/NoGo task, ii) smaller mean reduction in the number of total errors in executing the Groton maze learning task, and iii) larger mean increase in response time for the Stroop time ratio ((B-A)/A).

The change in CP usage had no significant effect on most of the changes in cognitive outcomes. However, the ‘increase’ in number of CP calls weekly group had smaller mean increase in accuracy in the detection task compared to those in the ‘no change or decrease’ group.

We did not find gender to be an effect modifier (results not shown).

## Discussion

This community-based longitudinal cohort study investigated whether the change in MPs/CPs in primary school children was associated with changes in cognitive function. The study contributes to the knowledge of psychological health and well-being associated with RF-EMF exposures in children, which has been part of the WHO high priority research agenda [8]. We found that number of calls and SMS weekly on MPs increased, whereas CP calls weekly remained unchanged from baseline to follow-up. Our results provide only limited evidence that change in the use of MPs/CPs in primary school children was associated with change in cognitive function.

The usage/ownership of MPs found in this sample of Australian children was low, but comparable to rates reported elsewhere. In the US, the use of MPs in children (aged 8 years and under) increased from 38 to 72% during 2011–2013 [21], In Europe, 33–83% of children aged up to 14 years [2, 4, 22], and 48% of Dutch children aged 5–6 years either used or owned a MP [14].

A relatively larger proportion of children used a CP, compared to a MP, which is similar to that found in the Netherlands [14]. The number MP and CP calls reported in our study is also comparable to that observed elsewhere [3, 14]. We found that a lower percentage of primary school children own/use a MP compared to ownership/usage rates reported in secondary school children [1, 11].

The follow-up analysis also showed that more children reported using/owning a MP than was reported by parents. A similar pattern was seen in our cross-sectional data [10]. This could be due to how the questions related to MP ownership and/or uses were asked and/or how they were understood by the parents and children. The potential implications of this for epidemiological studies have been discussed in the literature [1, 10, 23]. We suggest that the findings associated with the use of CP could be more accurate since the CP was used only at home and parents may thus have directly observed their child’s CP exposures.

The children had better cognitive performance at follow-up compared to baseline, which is expected as a natural part of development and has been reported in the literature [24]. In the cross-sectional analysis [10], the ‘more’ MP users had slower responses for the Go/NoGo task. However, the longitudinal analysis found that the ‘increase’ in MP calls group decreased their response time from baseline to follow-up more than the ‘no change’ group for this task. Furthermore, the cross-sectional findings observed that the ‘more’ CP (but not MP) users took longer when trying to overcome distraction (Stroop). In the longitudinal analysis no difference was seen in the CP groups, however the ‘increase’ in MP calls group had a larger mean increase in response time for a Stroop interference task ((B-A)/A) than the ‘no change’ group. Accuracy in executing the Groton maze learning task was increased in both MP groups from baseline to follow-up but less so in the ‘increase’ in MP calls group. Accuracy in the detection task increased for both CP groups from baseline to follow-up but less so for the ‘increase’ in CP calls group. The ‘increase’ in the CP calls group had lower accuracy in the detection task compared to those in the ‘no change or decrease’ group.

Our previous longitudinal study involving Australian secondary school children showed an increase in MP voice calls was associated with slower simple response time, but quicker working memory [1]. We did not find any similar results in the current study. A Swiss cohort study found that brain RF-EMF exposure among a ‘high’ exposed group (aged 12–17 years) was associated with a decrease in figural memory [11]. The memory tasks (i.e., one–back and one card learning) used in our study and the figural memory task were different. Therefore, caution is advised when comparing the findings. In the Netherlands, children aged 5–6 years showed that high CP users demonstrated slower response times in an inhibitory control and cognitive flexibility compared to non-users [14]. Although not identical, to the extent that the inhibitory control test and Go/NoGo task in our paper reflect the ‘inhibitory control’ cognitive process, and if CP/MP affects it, we would expect to see a similar pattern of results across the two studies. However, this was not the case: the Netherlands study reported slower reaction time in ‘high’ CP users, while we observed a faster reaction time amongst the group that increased their MP use.

Our study is the first to evaluate a longitudinal association of MP/CP use and change in cognition in a representative community-based sample of young children. We prospectively collected data at two time points. The study cohort consisted of a relatively large sample size, compared to those employed in provocation studies [25–27]. We assessed eight cognitive tasks, compared to the Swiss study (which only assessed two tasks) and the ABCD study (which assessed four tasks). This allowed us to look for potential effects on different cognitive domains as well as for whether consistent patterns of effects were present across multiple tasks (vs just in one task) within a cognitive domain.

The main limitation of the study was that phone exposure measures were self-reported. Our study was therefore likely to misclassify exposure and could provide biased findings. Objective exposure measurements using MP-based apps should be considered for prospective epidemiological studies, whenever possible [28]. Furthermore, the one-year follow-up time in our study may not be long enough to detect potential changes in cognitive outcomes associated with MPs/CPs exposure. A further follow-up may be more sensitive at determining whether a long-term usage of MPs and CPs affects cognition [29].

Another limitation of the study was that we did not assess motivation of the children while evaluating cognitive tasks. There may have been effects that were missed due to this. Furthermore, the high performance on the cognitive tasks may have reduced discriminatory power of the tasks and increased type II error, along with the misclassification of children’s use of MP/CP reported by parents/guardians.

## Conclusions

Our study shows that a larger proportion of children used CPs compared to MPs. The increase in MP usage was significantly associated with larger reduction in response time for response inhibition task, smaller reduction in the number of total errors in the Groton maze learning task, and larger increase in response time for a Stroop interference task. The increase in CP usage had no significant effect on the most of the changes in cognitive outcomes. Due to the small numbers of mobile and cordless phone calls, the observed changes in cognitive tasks could be pure chance findings. The use of mobile phones, as reported by children and parents was different. This should be taken into account in future studies by performing objective measurements of mobile phone exposures, whenever possible.

## Abbreviations

ABCD: Amsterdam born children and their development
CP: Cordless phone
ExPOSURE: Examination of psychological outcomes in students using radiofrequency devices
MP: Mobile phone
RF-EMF: Radiofrequency-electromagnetic field
SES: Socio-economic status
WHO: World Health Organization
